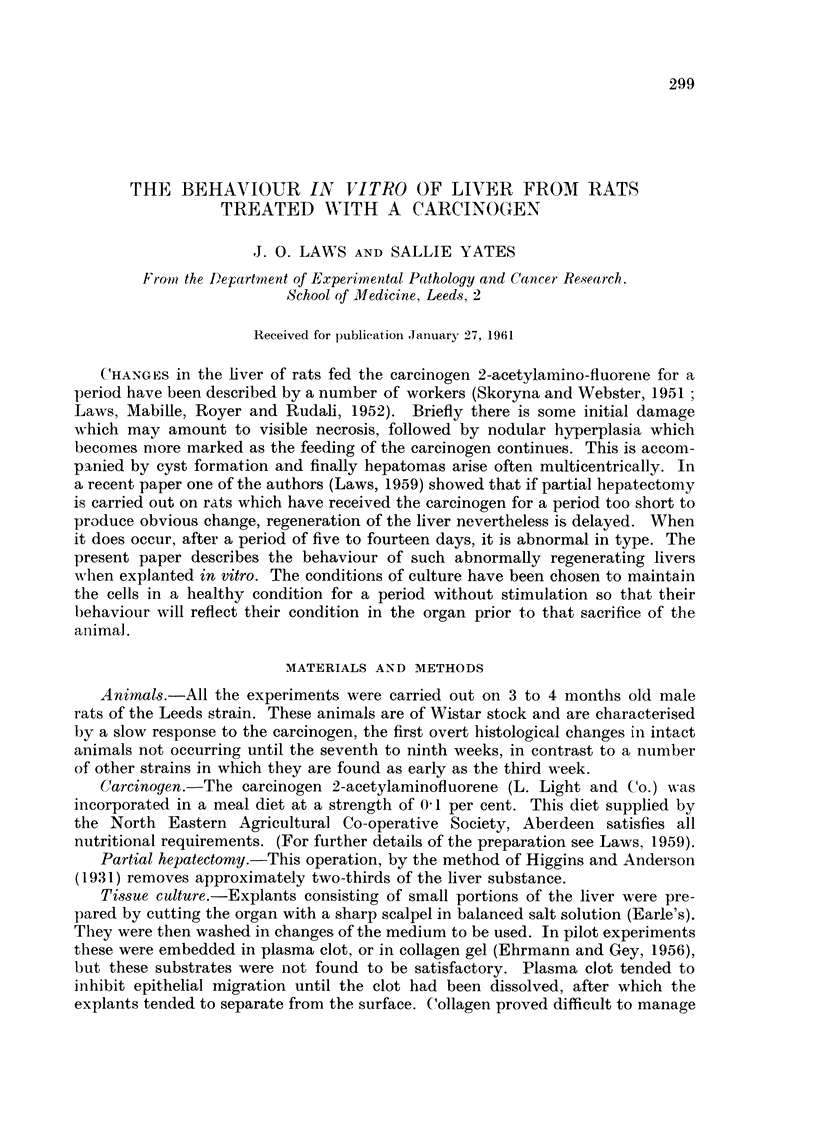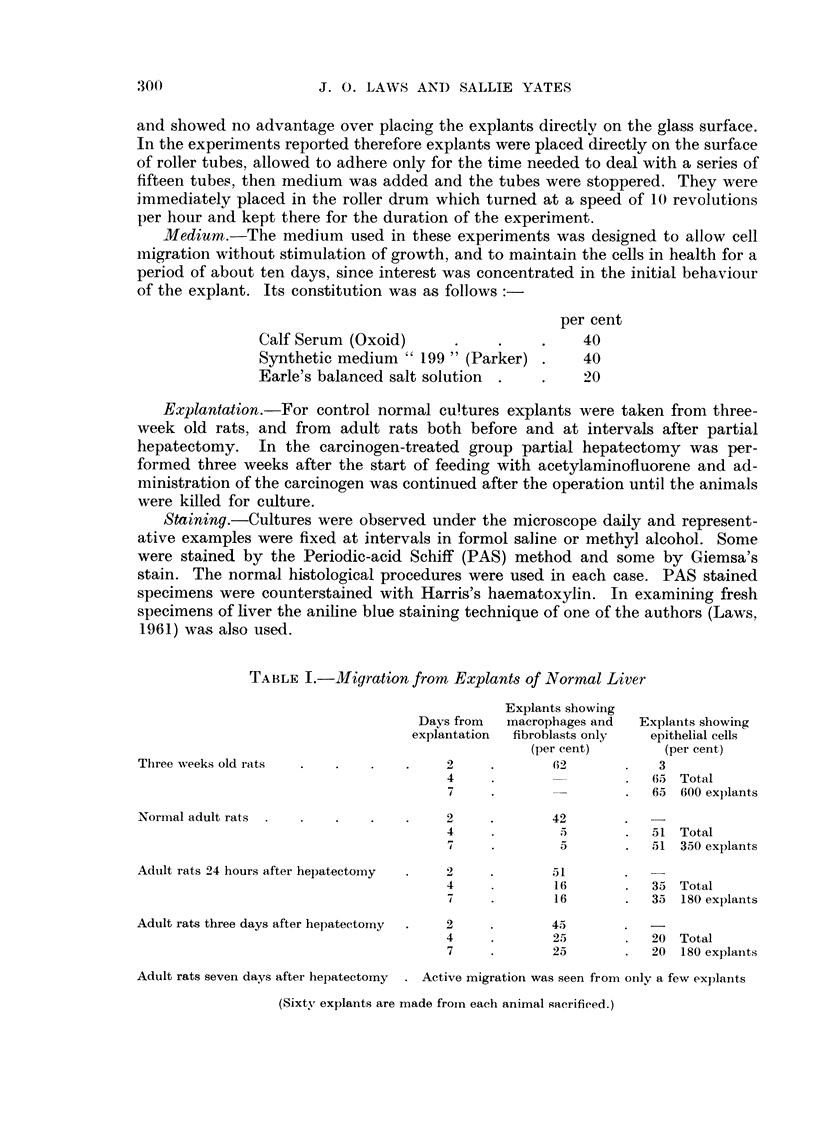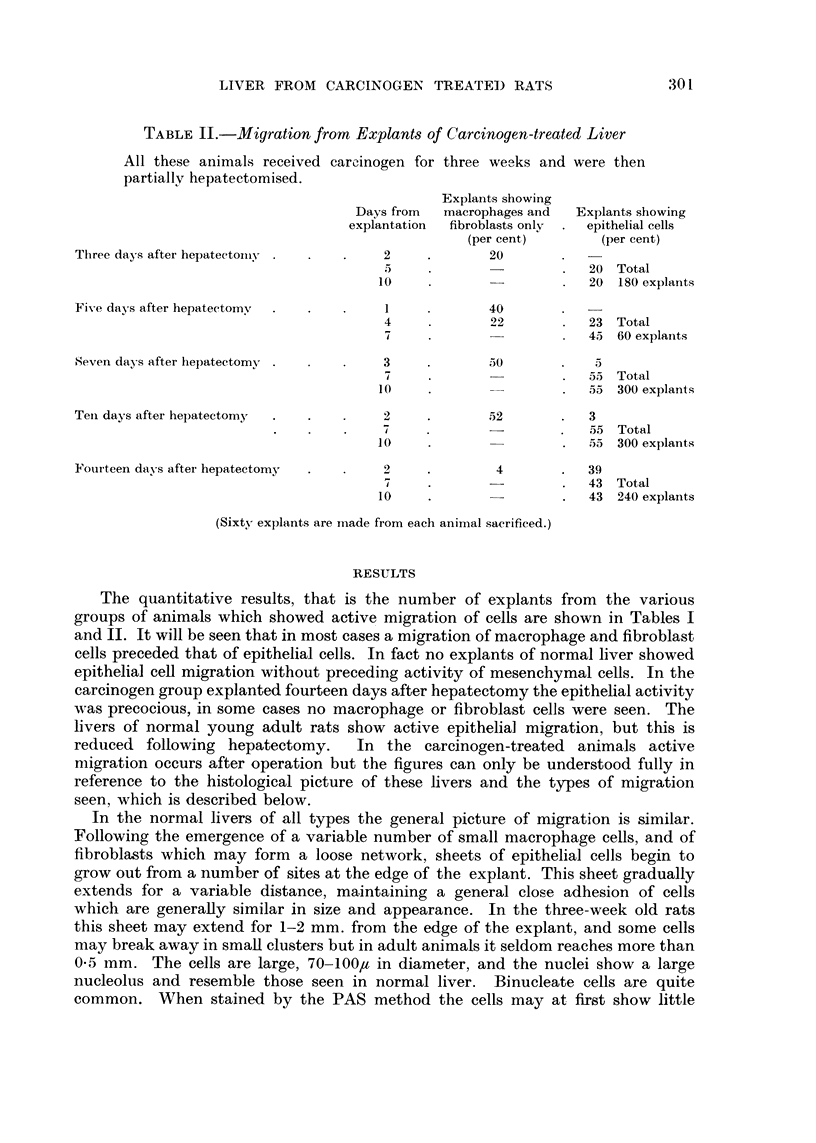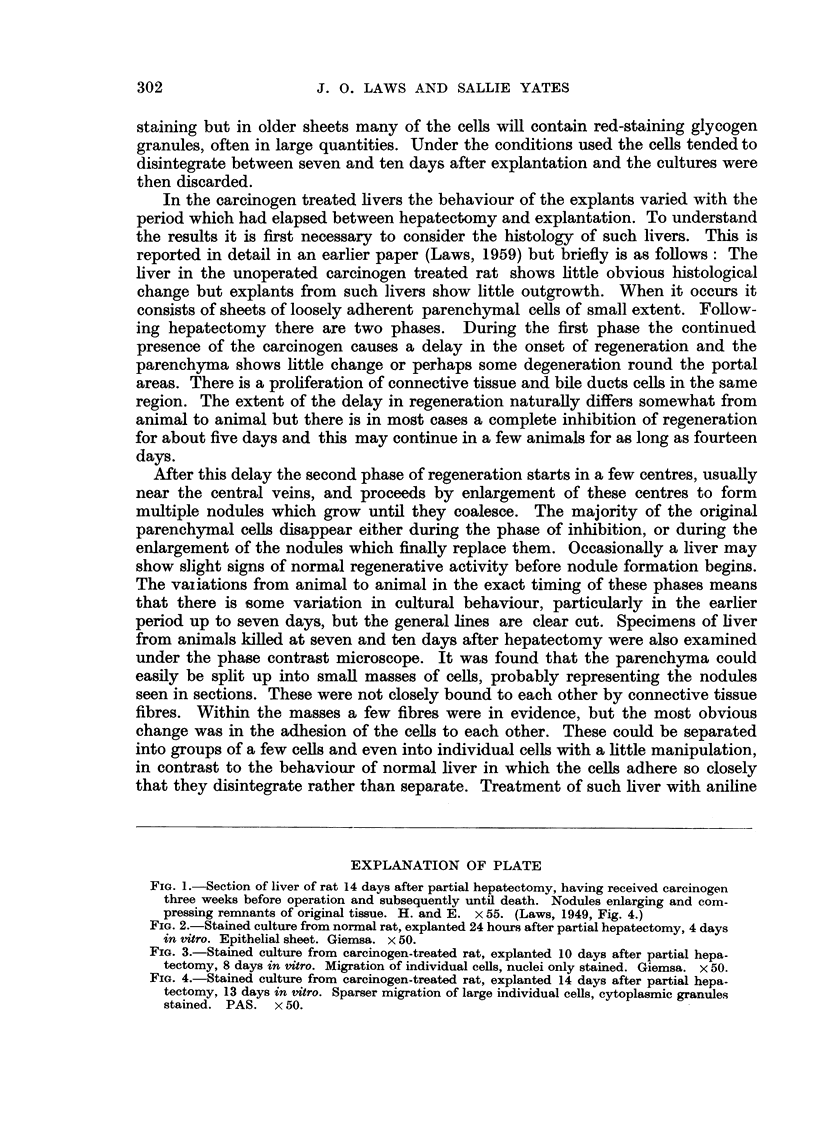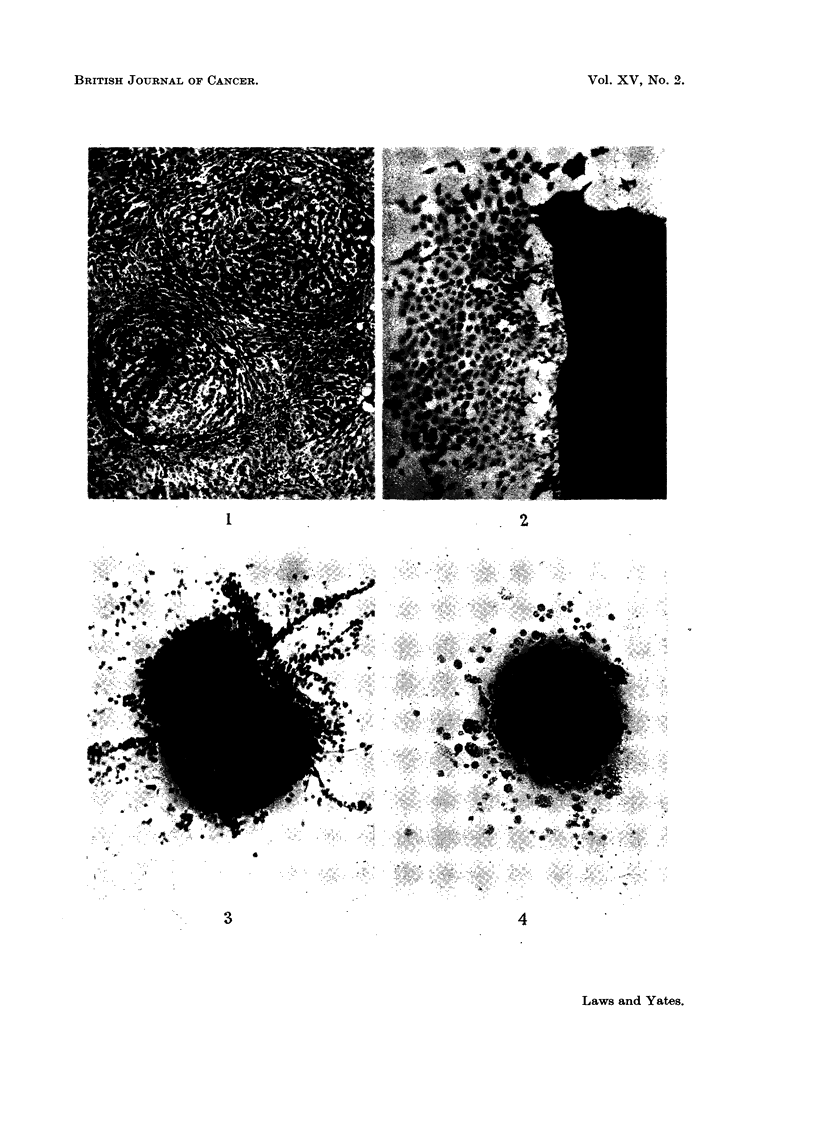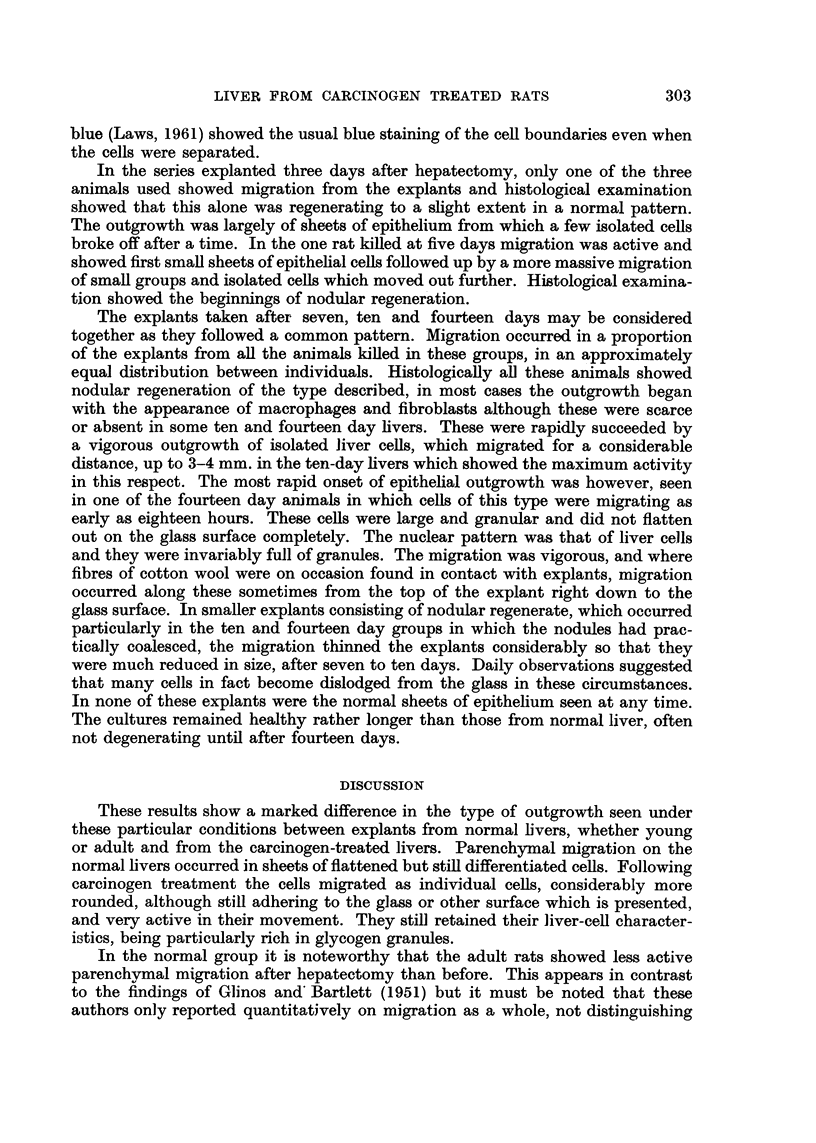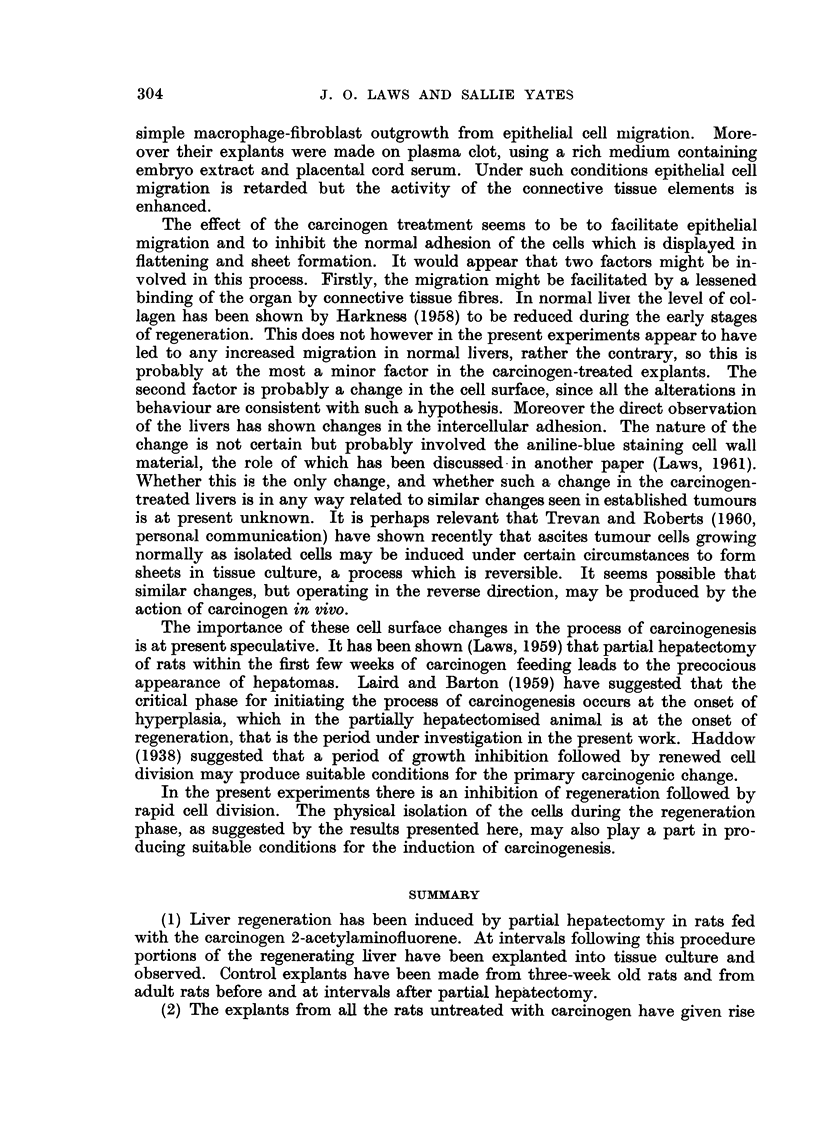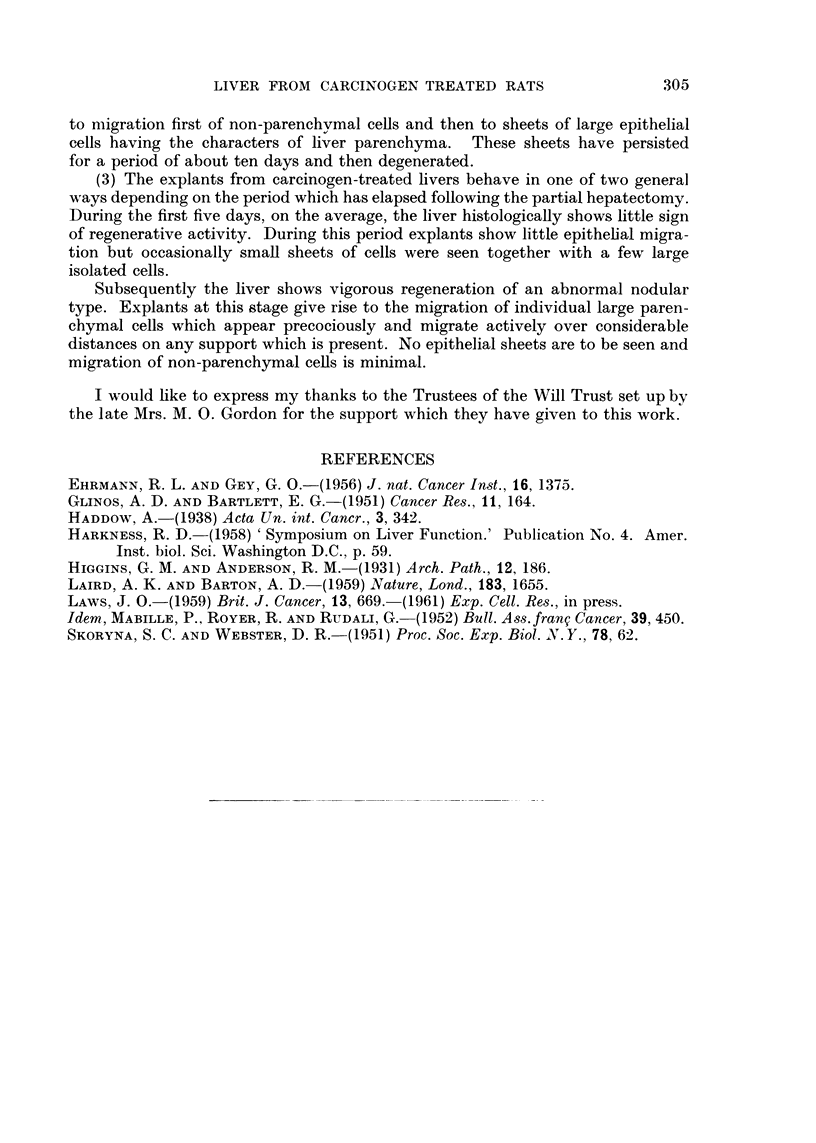# The Behaviour in vitro of Liver from Rats Treated with a Carcinogen

**DOI:** 10.1038/bjc.1961.37

**Published:** 1961-06

**Authors:** J. O. Laws, Sallie Yates

## Abstract

**Images:**


### THE BEHAVIOUR *IN VITRO* OF LIVER FROM RATS TREATED WITH A CARCINOGEN

J. O. LAWS AND SALLIE YATES

*From the Department of Experimental Pathology and Cancer Research, School of Medicine, Leeds, 2*

Received for publication January 27, 1961

CHANGES in the liver of rats fed the carcinogen 2-acetylamino-fluorene for a period have been described by a number of workers (Skoryna and Webster, 1951 ; Laws, Mabille, Royer and Rudali, 1952). Briefly there is some initial damage which may amount to visible necrosis, followed by nodular hyperplasia which becomes more marked as the feeding of the carcinogen continues. This is accompanied by cyst formation and finally hepatomas arise often multicentrically. In a recent paper one of the authors (Laws, 1959) showed that if partial hepatectomy is carried out on rats which have received the carcinogen for a period too short to produce obvious change, regeneration of the liver nevertheless is delayed. When it does occur, after a period of five to fourteen days, it is abnormal in type. The present paper describes the behaviour of such abnormally regenerating livers when explanted *in vitro.* The conditions of culture have been chosen to maintain the cells in a healthy condition for a period without stimulation so that their behaviour will reflect their condition in the organ prior to that sacrifice of the animal.

### MATERIALS AND METHODS

*Animals.*—All the experiments were carried out on 3 to 4 months old male rats of the Leeds strain. These animals are of Wistar stock and are characterised by a slow response to the carcinogen, the first overt histological changes in intact animals not occurring until the seventh to ninth weeks, in contrast to a number of other strains in which they are found as early as the third week.

*Carcinogen.*—The carcinogen 2-acetylaminofluorene (L. Light and Co.) was incorporated in a meal diet at a strength of 0·1 per cent. This diet supplied by the North Eastern Agricultural Co-operative Society, Aberdeen satisfies all nutritional requirements. (For further details of the preparation see Laws, 1959).

*Partial hepatectomy.*—This operation, by the method of Higgins and Anderson (1931) removes approximately two-thirds of the liver substance.

*Tissue culture.*—Explants consisting of small portions of the liver were prepared by cutting the organ with a sharp scalpel in balanced salt solution (Earle's). They were then washed in changes of the medium to be used. In pilot experiments these were embedded in plasma clot, or in collagen gel (Ehrmann and Gey, 1956), but these substrates were not found to be satisfactory. Plasma clot tended to inhibit epithelial migration until the clot had been dissolved, after which the explants tended to separate from the surface. Collagen proved difficult to manage and showed no advantage over placing the explants directly on the glass surface. In the experiments reported therefore explants were placed directly on the surface of roller tubes, allowed to adhere only for the time needed to deal with a series of fifteen tubes, then medium was added and the tubes were stoppered. They were immediately placed in the roller drum which turned at a speed of 10 revolutions per hour and kept there for the duration of the experiment.

*Medium.*—The medium used in these experiments was designed to allow cell migration without stimulation of growth, and to maintain the cells in health for a period of about ten days, since interest was concentrated in the initial behaviour of the explant. Its constitution was as follows :—

| | per cent |
|---|---|
| Calf Serum (Oxoid) . . . | 40 |
| Synthetic medium " 199 " (Parker) . | 40 |
| Earle's balanced salt solution . . | 20 |

*Explantation.*—For control normal cultures explants were taken from three-week old rats, and from adult rats both before and at intervals after partial hepatectomy. In the carcinogen-treated group partial hepatectomy was performed three weeks after the start of feeding with acetylaminofluorene and administration of the carcinogen was continued after the operation until the animals were killed for culture.

*Staining.*—Cultures were observed under the microscope daily and representative examples were fixed at intervals in formol saline or methyl alcohol. Some were stained by the Periodic-acid Schiff (PAS) method and some by Giemsa's stain. The normal histological procedures were used in each case. PAS stained specimens were counterstained with Harris's haematoxylin. In examining fresh specimens of liver the aniline blue staining technique of one of the authors (Laws, 1961) was also used.

TABLE I.—*Migration from Explants of Normal Liver*

| | Days from explantation | Explants showing macrophages and fibroblasts only (per cent) | Explants showing epithelial cells (per cent) | |
|---|---|---|---|---|
| Three weeks old rats | 2 | 62 | 3 | |
| | 4 | — | 65 | Total |
| | 7 | — | 65 | 600 explants |
| Normal adult rats | 2 | 42 | — | |
| | 4 | 5 | 51 | Total |
| | 7 | 5 | 51 | 350 explants |
| Adult rats 24 hours after hepatectomy | 2 | 51 | — | |
| | 4 | 16 | 35 | Total |
| | 7 | 16 | 35 | 180 explants |
| Adult rats three days after hepatectomy | 2 | 45 | — | |
| | 4 | 25 | 20 | Total |
| | 7 | 25 | 20 | 180 explants |
| Adult rats seven days after hepatectomy | Active migration was seen from only a few explants | | | |

(Sixty explants are made from each animal sacrificed.)

TABLE II.—*Migration from Explants of Carcinogen-treated Liver*

All these animals received carcinogen for three weeks and were then partially hepatectomised.

| | Days from explantation | Explants showing macrophages and fibroblasts only (per cent) | Explants showing epithelial cells (per cent) | |
|---|---|---|---|---|
| Three days after hepatectomy | 2 | 20 | — | |
| | 5 | — | 20 | Total |
| | 10 | — | 20 | 180 explants |
| Five days after hepatectomy | 1 | 40 | — | |
| | 4 | 22 | 23 | Total |
| | 7 | — | 45 | 60 explants |
| Seven days after hepatectomy | 3 | 50 | 5 | |
| | 7 | — | 55 | Total |
| | 10 | — | 55 | 300 explants |
| Ten days after hepatectomy | 2 | 52 | 3 | |
| | 7 | — | 55 | Total |
| | 10 | — | 55 | 300 explants |
| Fourteen days after hepatectomy | 2 | 4 | 39 | |
| | 7 | — | 43 | Total |
| | 10 | — | 43 | 240 explants |

(Sixty explants are made from each animal sacrificed.)

### RESULTS

The quantitative results, that is the number of explants from the various groups of animals which showed active migration of cells are shown in Tables I and II. It will be seen that in most cases a migration of macrophage and fibroblast cells preceded that of epithelial cells. In fact no explants of normal liver showed epithelial cell migration without preceding activity of mesenchymal cells. In the carcinogen group explanted fourteen days after hepatectomy the epithelial activity was precocious, in some cases no macrophage or fibroblast cells were seen. The livers of normal young adult rats show active epithelial migration, but this is reduced following hepatectomy. In the carcinogen-treated animals active migration occurs after operation but the figures can only be understood fully in reference to the histological picture of these livers and the types of migration seen, which is described below.

In the normal livers of all types the general picture of migration is similar. Following the emergence of a variable number of small macrophage cells, and of fibroblasts which may form a loose network, sheets of epithelial cells begin to grow out from a number of sites at the edge of the explant. This sheet gradually extends for a variable distance, maintaining a general close adhesion of cells which are generally similar in size and appearance. In the three-week old rats this sheet may extend for 1–2 mm. from the edge of the explant, and some cells may break away in small clusters but in adult animals it seldom reaches more than 0·5 mm. The cells are large, 70–100$\mu$ in diameter, and the nuclei show a large nucleolus and resemble those seen in normal liver. Binucleate cells are quite common. When stained by the PAS method the cells may at first show little staining but in older sheets many of the cells will contain red-staining glycogen granules, often in large quantities. Under the conditions used the cells tended to disintegrate between seven and ten days after explantation and the cultures were then discarded.

In the carcinogen treated livers the behaviour of the explants varied with the period which had elapsed between hepatectomy and explantation. To understand the results it is first necessary to consider the histology of such livers. This is reported in detail in an earlier paper (Laws, 1959) but briefly is as follows : The liver in the unoperated carcinogen treated rat shows little obvious histological change but explants from such livers show little outgrowth. When it occurs it consists of sheets of loosely adherent parenchymal cells of small extent. Following hepatectomy there are two phases. During the first phase the continued presence of the carcinogen causes a delay in the onset of regeneration and the parenchyma shows little change or perhaps some degeneration round the portal areas. There is a proliferation of connective tissue and bile ducts cells in the same region. The extent of the delay in regeneration naturally differs somewhat from animal to animal but there is in most cases a complete inhibition of regeneration for about five days and this may continue in a few animals for as long as fourteen days.

After this delay the second phase of regeneration starts in a few centres, usually near the central veins, and proceeds by enlargement of these centres to form multiple nodules which grow until they coalesce. The majority of the original parenchymal cells disappear either during the phase of inhibition, or during the enlargement of the nodules which finally replace them. Occasionally a liver may show slight signs of normal regenerative activity before nodule formation begins. The vaiiations from animal to animal in the exact timing of these phases means that there is some variation in cultural behaviour, particularly in the earlier period up to seven days, but the general lines are clear cut. Specimens of liver from animals killed at seven and ten days after hepatectomy were also examined under the phase contrast microscope. It was found that the parenchyma could easily be split up into small masses of cells, probably representing the nodules seen in sections. These were not closely bound to each other by connective tissue fibres. Within the masses a few fibres were in evidence, but the most obvious change was in the adhesion of the cells to each other. These could be separated into groups of a few cells and even into individual cells with a little manipulation, in contrast to the behaviour of normal liver in which the cells adhere so closely that they disintegrate rather than separate. Treatment of such liver with aniline

---

EXPLANATION OF PLATE

FIG. 1.—Section of liver of rat 14 days after partial hepatectomy, having received carcinogen three weeks before operation and subsequently until death. Nodules enlarging and compressing remnants of original tissue. H. and E. ×55. (Laws, 1949, Fig. 4.)

FIG. 2.—Stained culture from normal rat, explanted 24 hours after partial hepatectomy, 4 days *in vitro*. Epithelial sheet. Giemsa. ×50.

FIG. 3.—Stained culture from carcinogen-treated rat, explanted 10 days after partial hepatectomy, 8 days *in vitro*. Migration of individual cells, nuclei only stained. Giemsa. ×50.

FIG. 4.—Stained culture from carcinogen-treated rat, explanted 14 days after partial hepatectomy, 13 days *in vitro*. Sparser migration of large individual cells, cytoplasmic granules stained. PAS. ×50.

1

2

3

4

Laws and Yates.

blue (Laws, 1961) showed the usual blue staining of the cell boundaries even when the cells were separated.

In the series explanted three days after hepatectomy, only one of the three animals used showed migration from the explants and histological examination showed that this alone was regenerating to a slight extent in a normal pattern. The outgrowth was largely of sheets of epithelium from which a few isolated cells broke off after a time. In the one rat killed at five days migration was active and showed first small sheets of epithelial cells followed up by a more massive migration of small groups and isolated cells which moved out further. Histological examination showed the beginnings of nodular regeneration.

The explants taken after seven, ten and fourteen days may be considered together as they followed a common pattern. Migration occurred in a proportion of the explants from all the animals killed in these groups, in an approximately equal distribution between individuals. Histologically all these animals showed nodular regeneration of the type described, in most cases the outgrowth began with the appearance of macrophages and fibroblasts although these were scarce or absent in some ten and fourteen day livers. These were rapidly succeeded by a vigorous outgrowth of isolated liver cells, which migrated for a considerable distance, up to 3–4 mm. in the ten-day livers which showed the maximum activity in this respect. The most rapid onset of epithelial outgrowth was however, seen in one of the fourteen day animals in which cells of this type were migrating as early as eighteen hours. These cells were large and granular and did not flatten out on the glass surface completely. The nuclear pattern was that of liver cells and they were invariably full of granules. The migration was vigorous, and where fibres of cotton wool were on occasion found in contact with explants, migration occurred along these sometimes from the top of the explant right down to the glass surface. In smaller explants consisting of nodular regenerate, which occurred particularly in the ten and fourteen day groups in which the nodules had practically coalesced, the migration thinned the explants considerably so that they were much reduced in size, after seven to ten days. Daily observations suggested that many cells in fact become dislodged from the glass in these circumstances. In none of these explants were the normal sheets of epithelium seen at any time. The cultures remained healthy rather longer than those from normal liver, often not degenerating until after fourteen days.

### DISCUSSION

These results show a marked difference in the type of outgrowth seen under these particular conditions between explants from normal livers, whether young or adult and from the carcinogen-treated livers. Parenchymal migration on the normal livers occurred in sheets of flattened but still differentiated cells. Following carcinogen treatment the cells migrated as individual cells, considerably more rounded, although still adhering to the glass or other surface which is presented, and very active in their movement. They still retained their liver-cell characteristics, being particularly rich in glycogen granules.

In the normal group it is noteworthy that the adult rats showed less active parenchymal migration after hepatectomy than before. This appears in contrast to the findings of Glinos and Bartlett (1951) but it must be noted that these authors only reported quantitatively on migration as a whole, not distinguishing simple macrophage-fibroblast outgrowth from epithelial cell migration. Moreover their explants were made on plasma clot, using a rich medium containing embryo extract and placental cord serum. Under such conditions epithelial cell migration is retarded but the activity of the connective tissue elements is enhanced.

The effect of the carcinogen treatment seems to be to facilitate epithelial migration and to inhibit the normal adhesion of the cells which is displayed in flattening and sheet formation. It would appear that two factors might be involved in this process. Firstly, the migration might be facilitated by a lessened binding of the organ by connective tissue fibres. In normal liver the level of collagen has been shown by Harkness (1958) to be reduced during the early stages of regeneration. This does not however in the present experiments appear to have led to any increased migration in normal livers, rather the contrary, so this is probably at the most a minor factor in the carcinogen-treated explants. The second factor is probably a change in the cell surface, since all the alterations in behaviour are consistent with such a hypothesis. Moreover the direct observation of the livers has shown changes in the intercellular adhesion. The nature of the change is not certain but probably involved the aniline-blue staining cell wall material, the role of which has been discussed in another paper (Laws, 1961). Whether this is the only change, and whether such a change in the carcinogen-treated livers is in any way related to similar changes seen in established tumours is at present unknown. It is perhaps relevant that Trevan and Roberts (1960, personal communication) have shown recently that ascites tumour cells growing normally as isolated cells may be induced under certain circumstances to form sheets in tissue culture, a process which is reversible. It seems possible that similar changes, but operating in the reverse direction, may be produced by the action of carcinogen *in vivo*.

The importance of these cell surface changes in the process of carcinogenesis is at present speculative. It has been shown (Laws, 1959) that partial hepatectomy of rats within the first few weeks of carcinogen feeding leads to the precocious appearance of hepatomas. Laird and Barton (1959) have suggested that the critical phase for initiating the process of carcinogenesis occurs at the onset of hyperplasia, which in the partially hepatectomised animal is at the onset of regeneration, that is the period under investigation in the present work. Haddow (1938) suggested that a period of growth inhibition followed by renewed cell division may produce suitable conditions for the primary carcinogenic change.

In the present experiments there is an inhibition of regeneration followed by rapid cell division. The physical isolation of the cells during the regeneration phase, as suggested by the results presented here, may also play a part in producing suitable conditions for the induction of carcinogenesis.

### SUMMARY

(1) Liver regeneration has been induced by partial hepatectomy in rats fed with the carcinogen 2-acetylaminofluorene. At intervals following this procedure portions of the regenerating liver have been explanted into tissue culture and observed. Control explants have been made from three-week old rats and from adult rats before and at intervals after partial hepatectomy.

(2) The explants from all the rats untreated with carcinogen have given rise to migration first of non-parenchymal cells and then to sheets of large epithelial cells having the characters of liver parenchyma. These sheets have persisted for a period of about ten days and then degenerated.

(3) The explants from carcinogen-treated livers behave in one of two general ways depending on the period which has elapsed following the partial hepatectomy. During the first five days, on the average, the liver histologically shows little sign of regenerative activity. During this period explants show little epithelial migration but occasionally small sheets of cells were seen together with a few large isolated cells.

Subsequently the liver shows vigorous regeneration of an abnormal nodular type. Explants at this stage give rise to the migration of individual large parenchymal cells which appear precociously and migrate actively over considerable distances on any support which is present. No epithelial sheets are to be seen and migration of non-parenchymal cells is minimal.

I would like to express my thanks to the Trustees of the Will Trust set up by the late Mrs. M. O. Gordon for the support which they have given to this work.

### REFERENCES

Ehrmann, R. L. and Gey, G. O.—(1956) *J. nat. Cancer Inst.*, **16**, 1375.
Glinos, A. D. and Bartlett, E. G.—(1951) *Cancer Res.*, **11**, 164.
Haddow, A.—(1938) *Acta Un. int. Cancr.*, **3**, 342.
Harkness, R. D.—(1958) ' Symposium on Liver Function.' Publication No. 4. Amer. Inst. biol. Sci. Washington D.C., p. 59.
Higgins, G. M. and Anderson, R. M.—(1931) *Arch. Path.*, **12**, 186.
Laird, A. K. and Barton, A. D.—(1959) *Nature, Lond.*, **183**, 1655.
Laws, J. O.—(1959) *Brit. J. Cancer*, **13**, 669.—(1961) *Exp. Cell. Res.*, in press.
*Idem*, Mabille, P., Royer, R. and Rudali, G.—(1952) *Bull. Ass. franç Cancer*, **39**, 450.
Skoryna, S. C. and Webster, D. R.—(1951) *Proc. Soc. Exp. Biol. N.Y.*, **78**, 62.